# Real-world study of the leakage of two types of infusion bags in multicenter pharmacy intravenous admixture service (PIVAS)

**DOI:** 10.3389/fphar.2023.1273020

**Published:** 2023-10-06

**Authors:** Yanchao Yin, Wei Fu, Wenhua Liu, Feie Li, Xuepeng Gong, Dong Liu, Juan Li

**Affiliations:** ^1^ Department of Pharmacy, Tongji Hospital, Tongji Medical College of Huazhong University of Science and Technology, Wuhan, China; ^2^ Clinical Research Center, Tongji Hospital, Tongji Medical College of Huazhong University of Science and Technology, Wuhan, China

**Keywords:** pharmacy intravenous admixture service (PIVAS), non-polyvinyl chloride (non-PVC) infusion bags, upright polypropylene infusion bags, leakage, real-world study

## Abstract

**Background:** This study sought to analyze the leakage rate, economic loss caused by leakage, leakage reasons, and usage of upright polypropylene infusion bags and non-polyvinyl chloride (PVC) infusion bags, two types of closed intravenous infusion containers used in pharmacy intravenous admixture service (PIVAS), to improve the product quality of drug infusion packaging materials, reduce drug and clinical economic losses, and reduce the safety hazards of medication.

**Method:** A real-world study was used to collect statistics for these infusion containers. The study was conducted in 21 hospitals in China from September to December 2022. Upright polypropylene infusion bags or non-PVC infusion bags in PIVAS of these 21 hospitals were chosen as the research material.

**Results:** In total, 2,349,899 upright polypropylene infusion bags and 3,301,722 non-PVC infusion bags were collected. Eleven cases of upright polypropylene infusion bag leakage occurred (with a the leakage rate of 0.05‱), and 394 cases of non-PVC infusion bag leakage occurred (with a leakage rate of 1.19‱). The leakage rate of non-PVC infusion bags was significantly higher than that of upright polypropylene infusion bags (*p* < 0.01). The main reason for leakage in upright polypropylene infusion bags was sharp objects such as glass fragments or aluminum caps piercing the bag. The main reason for leakage in non-PVC infusion bags was squeezing, stacking, and uneven arrangement that causes folding of edges. For non-PVC bags, additional reasons for leakage included leakage at the nozzle joint, excessive manual or machine throwing force, and excessive dosage. The economic loss of upright polypropylene infusion bags was 1,116.56 CNY. The economic loss of non-PVC infusion bags was 32,210.86 CNY.

**Conclusion:** Based on real-world study data on the leakage of upright polypropylene infusion bags and non-PVC infusion bags in multicenter PIVAS, it can be concluded that the leakage rates of upright polypropylene infusion bags are significantly lower than those of non-PVC infusion bags in PIVAS, and the economic losses due to upright polypropylene infusion bags are lower than those due to non-PVC infusion bags in PIVAS. Therefore, we can infer that upright polypropylene infusion bags are superior to non-PVC infusion bags.

## Introduction

Intravenous infusion therapy is a commonly used drug delivery route, especially for hospitalized patients. It has accessible drug delivery routes, simple operation and quick effects. Intravenous infusion therapy is a type of treatment that conforms to China’s national conditions and is widely used in clinics. According to the statistics of the National Health Commission of the People’s Republic of China (PRC), the utilization rate of intravenous infusion among hospitalized patients in China is approximately 80%–90%, and this utilization rate has been stable in recent years (Zuo et al., 2023). According to the report *“Study on the Current Situation of the Regular Infusion Industry and Related Policies (2023)”* from the China National Pharmaceutical Industry Information Center, the amount of regular infusion (such as glucose or sodium chloride) used in China in 2019 was approximately 6 billion bottles/bags. With the development of the pharmaceutical industry and the continuous improvement of clinical demands, intravenous infusion systems have been gradually upgraded, and open systems have been replaced with closed systems (Maki et al., 1974). A closed intravenous infusion system means that the infusion drug containers are fully self-folding and do not require or use an external vent to empty the infusion solution, which is safer and more economical than an open system (Tarricone et al., 2010). Closed intravenous infusion systems have been widely used globally, but there is still a gap in the penetration rate in China (Maki et al., 2011). However, with the popularization of clinical safety infusion and the normalization of the prevention and control of COVID-19, closed intravenous infusion systems have received further attention and recognition. In most developed countries, closed intravenous infusion containers are dominated by PVC infusion bags and non-PVC infusion bags. For example, in Europe and the United States, the usage rate of PVC infusion bags and non-PVC infusion bags is 70–90% (Pourroy et al., 2010). In China, in addition to PVC infusion bags and non-PVC infusion bags, upright polypropylene infusion bags are widely used. However, due to the risk of bis(2-ethylhexyl) phthalate (DEHP) dissolution from PVC material, the National Medical Products Administration has stopped the approval of the PVC bag production line since 2000 (Kim et al., 2005; Erythropel et al., 2014). Currently, closed intravenous infusion containers in China are non-PVC infusion bags and upright polypropylene infusion bags, which are both widely used in clinics.

Because infusion containers have direct contact with drugs, their material quality directly affects the safety of patients, and their packaging form affects the convenience of clinical usage. Regular infusion has a special function as a solvent to admix therapeutic drugs, which has a significant impact. Different infusion containers may differ in quality and form due to differences in their main materials, additive formulation and production processes (Tchiakpé et al., 1995; Mattiazzi et al., 2019). Currently, research on the differences in infusion containers mainly focuses on drug compatibility (Mohr and Kramer, 2022), and most studies have compared PVC infusion bags with non-PVC infusion bags (Rigge and Jones, 2005). For example, studies have reported that PVC infusion bags have certain adsorption for a variety of drugs, which affects the therapeutic effect; therefore, PVC infusion bags should be avoided for clinical use (Sürmelioğlu et al., 2021). However, there is no difference in drug compatibility between non-PVC infusion bags and upright polypropylene infusion bags (Li H. et al., 2019). Previous studies lack guidance on the use of intravenous infusion containers in China. The leakage of regular infusion occasionally occurs in clinical practice, which not only leads to drug loss but also affects operation efficiency and increases workload. In addition, if a leaking infusion is accidentally used in clinical practice, it may cause serious medication risks. There are many factors that may cause leakage, and attention has been given to the influence of external factors, such as puncture by sharp objects during operation, excessive throwing force, and falling damage (Li S. H. et al., 2019). In long-term clinical use in China, it was found that the leakage of non-PVC infusion bags was higher than that of upright polypropylene infusion bags. This phenomenon may be related to the characteristics of the different containers. However, no systematic real-world studies have focused on the difference in leakage rates between the two kinds of infusion containers, the causes of leakage, and the economic losses caused by leakage. Therefore, the leakage of regular infusion containers is worthy of attention and research.

Since the world’s first intravenous admixture service center was established in 1969, the centralized aseptic configuration of intravenous medications has spread worldwide. Since 1980, it has been vigorously developed in some countries such as the United States and Europe (Mi et al., 2018). Although they have different names, such as intravenous admixture service (IVAS) in the United States and Australia, Central intravenous admixture service (CIVAS) in Europe, their functions are similar (Hedlund et al., 2017; Jessurun et al., 2022). However, the intravenous admixture service in China was established based on the development of pharmacy, thus it is called pharmacy intravenous admixture service (PIVAS) (Chen et al., 2021). PIVAS is a hospital-based comprehensive and technical pharmaceutical department, and an important branch in Chinese hospitals (Liao et al., 2020). Because PIVAS can improve occupational protection and ensure the safety and effectiveness of intravenous infusions, it has been widely promoted in China. The first PIVAS in China was established in 1999 in Shanghai Jing’an District Central Hospital, and more than 1,100 hospitals have since established PIVAS across the country (Yang et al., 2020). The Chinese government has issued relevant policies and standards to encourage the establishment and development of PIVAS (State Pharmacopoeia Committee, 2020). According to the Regulations on the Administration of Pharmaceutical Affairs in Medical Institution, medical institutions should establish PIVAS for antineoplastic drugs and total parenteral nutrition (TPN) based on clinical demands. Guidelines for the construction and management of PIVAS were first published in 2010 and were issued by the National Health Commission in 2021 (National Health Commission of People’s Republic of China, 2021). The establishment of PIVAS provides a scientific means of dispensing intravenous infusion and is an inevitable development trend. The centralized admixture of intravenous drugs plays an obvious role in improving the quality of infusion and promoting rational drug use. For example, the establishment of normative technical operating procedures for admixing and dispensing standards and the addition of checking measures to ensure the quality of finished infusion products play key roles in reducing leakage caused by nonstandard operation. Currently, the main research directions for PIVAS are the training of staff (Ni et al., 2021), construction costs and charges (Yang et al., 2020), information-intelligence technologies (Wang et al., 2022), and occupational exposure (Zhang et al., 2016; Roussel et al., 2019). The quality of infusion is also an important factor in PIVAS. Therefore, the problem of leakage of intravenous infusion containers, which may cause drug safety and economic problems, has also become a vital factor that cannot be ignored.

This study took regular infusion as the research object and systematically compared the difference in the leakage rates of two kinds of closed intravenous infusion containers in PIVAS as well as the causes of leakage and the economic losses due to leakage in a real-world study. We hope our work can provide recommendations for container selection for intravenous infusion in China, improve the quality of infusion products, reduce drug and clinical economic losses, and reduce the safety risk of infusion products in clinics.

## Materials and methods

### Materials

Non-PVC infusion bags or upright polypropylene infusion bags were chosen as the research materials. Non-PVC infusion bags are made of non-PVC composite film by heat welding, and the infusion cover and the bag body are connected by heat welding. Upright polypropylene infusion bags are made of polypropylene pellets by a hot-melt process, the bag body is formed in one piece without welding points, and the bag body and infusion cover are connected by hot-melt welding.

### Study design

This multicenter PIVAS study was conducted in 21 hospitals in China from September to December 2022. In this study, PIVAS of 21 regional tertiary hospitals in China were included to ensure that the operations were standardized and the staff had received professional training. And all research centers are constructed and accepted as qualified centers in accordance with the National Health Commission’s Guidelines for the Construction and Management of PIVAS (National Health Commission of PRC, 2021). Upright polypropylene infusion bags or non-PVC infusion bags in the PIVAS of these 21 hospitals were chosen as the research material. Pharmacists and nurses working in these PIVAS were selected as participants to report leakage and complete questionnaires. While designing the study, we investigated the number of infusions used in each study center to try to ensure that the quantities of the two types of infusion containers were equal. However, due to the divergent requirements for COVID-19 epidemic prevention in different provinces in China during the study cycle, there were some deviations in the quantities.

### Data collection

The data collection method was a web-based questionnaire system and Excel tables, through which relevant information about the leakage of infusion containers was collected and summarized. The daily use of regular infusion drugs was registered in Excel tables, and this information was summarized and uploaded to e-mail (lyl_tjh.com) once a week from September to December. Each center confirmed a project leader who was responsible for all data content. The data content included the following. Basic Information Table: Basic hospital information, filling persons, and regular infusion drug information; Weekly statistical table: The quantity of regular infusion drugs every day, which was uploaded to e-mail once a week; Leakage report: leakage information such as basic leakage information, leakage stages, leakage reasons, costs, and leakage images, etc; Satisfaction questionnaire: Operational convenience survey, fall resistance survey, impact of leakage on work efficiency survey, etc. Data collection required timeliness, accuracy, completeness, standardization, and authenticity.

First of all, all research centers were required to fill out Basic Information Table before the study. Then, the daily usage of regular infusion bags was registered in Weekly statistical table every day, and summarized once a week. The summary was sent to the e-mail from September to December. During this period, if leakage occurs, a Leakage report needs to be filled out. Finally, we invited all participants in the study to fill out a Satisfaction questionnaire.

### Data analysis

SPSS 24.0 statistical software was used. The statistical analysis content included the actual selected quantity, evaluation of fluid leakage, safety analysis, and satisfaction analysis. The *χ* ^2^ test was used to compare the count data, and *p* < 0.05 was considered statistically significant. Cronbach’s alpha coefficient was used to evaluate the questionnaire. The data for the satisfaction analysis were tested by the Kolmogorov‒Smirnov test. Non-normal distribution was described by the median (interquartile distance) [M(Q1,Q3)], and the Mann‒Whitney U test was used for statistical methods; *p* < 0.05 was considered statistically significant.

## Results

### Basic information of participants

A total of 21 tertiary hospitals participated in the study (Table 1). Regarding the geographic location of the participants, 38.1% (8/21) of the participants were located in central China, 38.1% (8/21) in eastern China, and 23.8% (5/21) in western China. The numbers of staff ranged from 4 to 90, and the approximately daily admixture quantities ranged from 160 to 13000 and were related to the size of PIVAS. A total of 66.7% of centers were charged for common drugs and antibiotic drugs, with a range of 4.75 ± 2.99 CNY and 5.54 ± 3.46 CNY. All research centers were charged for antineoplastic drugs, with a range of 28.51 ± 17.54 CNY. The types of regular infusion fluids included 0.9% sodium chloride injection, 5% glucose injection, 10% glucose injection, and glucose sodium chloride injection.

**TABLE 1:** Basic information of 21 multicenter PIVAS.

Name	Location	Number of staff	Number of daily admixture	Admixture Charge (CNY)		
				Common drug	Antibiotic drug	Antineoplastic drug
Tongji Hospital, Tongji Medical College of HUST	Centre	4	160	9	10	54
Renmin Hospital of Wuhan University	Centre	8	392	9	10	54
Zhongnan Hospital of Wuhan University	Centre	27	3200	9	10	54
Wuhan Hospital of Traditional Chinese And Western Medicine	Centre	24	2685	9	10	54
Henan Cancer Hospital	Centre	90	13000	2	2	35
The First Affiliated Hospital of Zhengzhou University	Centre	36	2100	2	2	35
The First Affiliated Hospital of Nanchang University	Centre	54	5124	—	—	40
The Third XiangYa Hospital of Central South University	Centre	49	8000	3	3	12
Shanghai Tenth People’s Hospital	East	31	3100	—	—	10
Zhongshan Hospital, Fudan University	East	35	4000	—	—	10
Binzhou Medical University Hospital	East	44	5000	4.5	4.5	40
Qilu Hospital of Shandong University	East	66	11000	4.5	6.5	40
The First Affiliated Hospital, Zhejiang University School of Medicine	East	68	11000	—	—	16.9
The First Affiliated Hospital of Ningbo University	East	39	4000	—	—	16.9
The First Hospital of China Medical University	East	33	1390	—	—	11
The First Affiliated Hospital of Xiamen University	East	60	8000	1	1	46
Affiliated Hospital of North Sichuan Medical College	West	30	2600	2.5	2.5	7
The Third People’s Hospital of Chengdu	West	14	1600	3	3	8
The First Affiliated Hospital of Xinjiang Medical University	West	54	11000	—	—	14
The Affiliated Hospital of Guizhou Medical University	West	30	3300	3	5	18
The First People’s Hospital of Yunnan Province	West	43	4000	5	8	23

### Comparison of leakage of two types of infusion containers

From September 1 to December 31, 2022, a total of 2,349,899 upright polypropylene infusion bags and 3,301,722 non-PVC infusion bags were collected in this study (Table 2). In total, 11 cases of upright polypropylene infusion bag leakage occurred, with a leakage rate of 0.05‱ (‱ stands for “one per 10,000”). Additionally, 394 cases of non-PVC infusion bag leakage occurred, with a leakage rate of 1.19‱. The leakage rate of non-PVC infusion bags was significantly higher than that of upright polypropylene infusion bags.

**TABLE 2:** Analysis of the leakage of upright polypropylene infusion bags and non-PVC infusion bags.

Leakage detection scenes	Upright polypropylene			None-PVC			*χ* ^2^	*p*
	Number of leaky bags	Number of used bags	Leakage rate(‱)	Number of leaky bags	Number of used bags	Leakage rate(‱)		
All	11	2349899	0.05	394	3301722	1.19	251.84	<0.001
Pre-PIVAS	0	2349899	0	159	3301722	0.48	113.17	<0.001
In-PIVAS	5	2349899	0.02	167	3301563	0.51	105.91	<0.001
Post-PIVAS	6	2349894	0.03	68	3301396	0.21	34.134	<0.001

**‱** stands for “one per 10,000”.

The leakage detection scenes were divided into three categories: pre-PIVAS (storage stage, leakage of infusion fluids detected upon removal of carton overwrap), in-PIVAS (leakage of infusion fluids detected during PIVAS admixture), and post-PIVAS (clinical stage, leakage of infusion fluids detected after admixture and packing out of PIVAS). In the pre-PIVAS stage, leakage occurred only in non-PVC infusion bags; 159 cases of leakage occurred, with a leakage rate of 0.48‱. In PIVAS, 5 cases of upright polypropylene infusion bag leakage occurred, with a leakage rate of 0.02‱, and 167 cases of non-PVC infusion bag leakage occurred, with a leakage rate of 0.51‱. In the post-PIVAS stage, 6 cases of upright polypropylene infusion bag leakage occurred, with a leakage rate of 0.03‱, and 68 cases of non-PVC infusion bag leakage occurred, with a leakage rate of 0.21‱. Based on these results, we found that in pre-PIVAS, in PIVAS, or in post-PIVAS, the leakage rate of non-PVC infusion bags was seemingly higher than that of upright polypropylene infusion bags.

### Analysis of leakage reasons

This study identified 10 common reasons that might lead to leakage, including sharp objects such as glass fragments or aluminum caps piercing the bag; excessive manual or machine throwing force; falling; squeezing, stacking, and uneven arrangement causing folding of edges; excessive dosage; and other reasons (Table 3). The occurrence of leakage could be caused by one or several factors. The causes of leakage in upright polypropylene infusion bags were divided into four categories. The main reason was sharp objects piercing the bag, accounting for 72.73%. The proportion of excessive manual or machine throwing force and falling was consistent, accounting for 27.27%. There was also one case of leakage caused by squeezing, stacking, and uneven arrangement causing folding of edges. There are many reasons for leakage of non-PVC infusion bags, mainly squeezing, stacking, and uneven arrangement causing folding of edges, which accounted for 39.56% of leakage. There were also 155 cases of leakage caused by transportation before unpacking the carton, accounting for 39.34%. The other reasons were leakage at the nozzle joint, excessive manual or machine throwing force, and sharp objects such as glass fragments or aluminum caps piercing the bag, accounting for 10.41%, 6.85%, and 6.6%, respectively. In addition, there were problems with excessive dosage (2.28%), falling (2.03%), leakage at the joint (1.78%), valve port quality issues (1.52%), and other issues (4.82%).

**TABLE 3:** Analysis of the causes of leakage of upright polypropylene infusion bags and non-PVC infusion bags.

Type	Cause of leakage	Case	Proportion of total leakage cases (%)	Proportion of total number of used bags (‱)
Upright polypropylene	Sharp objects such as glass fragments or aluminum caps pierce the bag	8	72.73	0.034
	Excessive manual or machine throwing force	3	27.27	0.013
	Falling	3	27.27	0.013
	Squeezing, stacking, and uneven arrangement cause folding of edges	1	9.09	0.004
Non-PVC	Squeezing, stacking, and uneven arrangement cause folding of edges	156	39.59	0.472
	Leakage caused by transportation before unpacking the carton	155	39.34	0.469
	Leakage at the nozzle joint	41	10.41	0.124
	Excessive manual or machine throwing force	27	6.85	0.081
	Sharp objects such as glass fragments or aluminum caps pierce the bag	26	6.60	0.079
	Excessive dosage	9	2.28	0.027
	Falling	8	2.03	0.024
	Leakage at the joint	7	1.78	0.021
	Valve port quality issues	6	1.52	0.018
	Other reasons	19	4.82	0.058

**‱** stands for “one per 10,000”.

### Analysis of the economic loss due to leakage

The economic loss caused by leakage is closely related to whether it is admixed and the types of admixed drugs. These data present only the objective data of this study (Table 4). In this study, 11 upright polypropylene infusion bags leaked with a total economic loss of 1116.56 CNY, and 394 non-PVC infusion bags leaked with a total economic loss of 3,221,086 CNY.

**TABLE 4:** Analysis of economic loss of leakage.

Classification	Items	Upright polypropylene bags	Non-PVC bags
Overall situation	Quantity of leakage	11	394
	Total economic loss of leakage	1116.56	32210.86
Not admixed	Quantity of leakage	0	239
	Total economic loss of leakage	0	1140.06
Admixed	Amount of common drug leakage	7	129
	Economic loss of common drug leakage	554.47	7023.16
	Amount of antibiotic drug leakage	3	21
	Economic loss of antibiotic drug leakage	444.62	2584.22
	Amount of antineoplastic drug leakage	1	5
	Economic loss of antineoplastic drug leakage	117.47	21463.42
	Average leakage economic loss	193.90	
	Average mixing and processing time	11.97	

These infusion bags were divided into two types: one was not admixed with other drugs, and the other was already admixed. When the infusion bag was not admixed, only the cost of regular infusion was calculated. When the regular infusion was admixed, the cost of regular infusion, admixed drugs, consumables, and expenses of labor were calculated. For the non-admixed type, no upright polypropylene infusion bags leaked, while 239 non-PVC infusion bags leaked, resulting in a total economic loss of 1,140.06 CNY. For the already admixed type, there were 7 cases of common drug leakage from upright polypropylene infusion bags with an economic loss of 554.47 CNY, 3 cases of antibiotic drug leakage with an economic loss of 444.62 CNY, and 1 case of antineoplastic drug leakage with an economic loss of 117.47 CNY. For non-PVC infusion bags, there were 129 cases of common drug leakage with an economic loss of 7,023.16 CNY, 21 cases of antibiotic drug leakage with an economic loss of 2,584.22 CNY, and 5 cases of antineoplastic drugs with an economic loss of 21,463.42 CNY. Because the actual economic and time loss after leakage is related to the type of drugs used, the average value of the impact caused by leakage is not closely related to containers, so the two types of containers were combined. In total, there were 166 cases of leakage in the two types of containers, resulting in an average economic loss of 193.9 CNY and an average mixing and processing time of 11.97 min.

### Analysis of satisfaction questionnaire on infusion containers

The basic information of PIVAS staff includes professional titles and work experiences. Of the staff members, 141 were junior pharmacists, 94 were senior pharmacists, 13 were chief pharmacists, and 11 had other positions. A total of 43.24% of the staff had worked for more than 10 years, 29.73% of the staff had worked for 0–5 years, and 27.03% of the staff had worked for 5–10 years. The Cronbach’s *α* of the upright polypropylene bag survey was 0.617, while the Cronbach’s *α* of the non-PVC bag survey was 0.677. The satisfaction questionnaire had good reliability and construct validity. In terms of the operational convenience survey, there was no significant difference between upright polypropylene infusion bags and non-PVC infusion bags. However, the difference was statistically significant in terms of fall resistance, pressure resistance, leakage economy, and time loss, indicating that upright polypropylene infusion bags are better to use (Table 5). This evaluation refers only to the experiential score of the two infusion containers during a long working period. A higher score indicates better use of the infusion container.

**TABLE 5:** Satisfaction score statistics for upright polypropylene bags and non-PVC bags.

Items	Upright polypropylene	Non-PVC	z	*p*
Operational convenience score	4(4,5)	4(4,5)	−0.863	0.388
Fall and pressure resistance score	4(4,5)	3(3,4)	−9.180	<0.001
Leakage economy and time loss score	4(3,4)	3(2,4)	−4.255	<0.001

**FIGURE 1:**
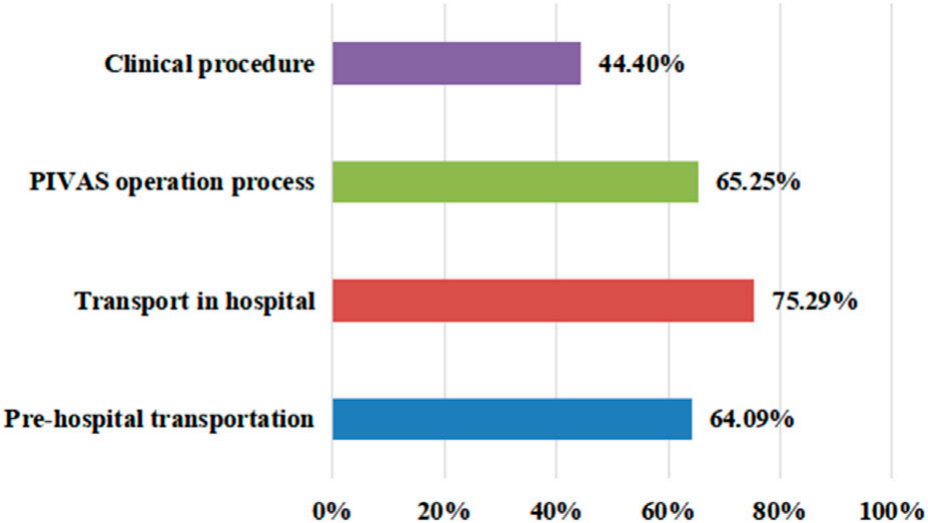
Results of the investigation of the common occurrence of leakage in clinical work.

**FIGURE 2:**
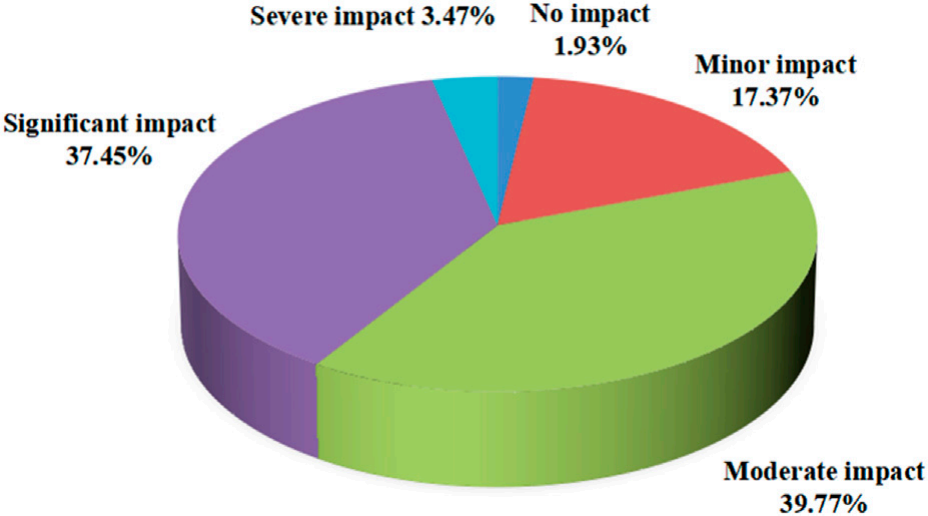
Effect of leakage on work efficiency.

The survey on the conditions of leakage in clinical work showed that 75.29% of staff members chose transportation in hospitals, with a minimum of 44.4% choosing clinical operations (Figure 1). However, in the survey on the impacts of leakage on work efficiency, 1.93% of staff members believed that it did not cause an impact, while 39.77% of employees believed there was a moderate impact; 37.45% of employees believed that the impact was significant, and 3.47% of employees believed that the impact was severe (Figure 2).

## Conclusion

It can be concluded that the leakage rates of upright polypropylene infusion bags are statistically significantly lower than those of non-PVC infusion bags in PIVAS, and economic losses from upright polypropylene infusion bags are lower than those of non-PVC infusion bags in PIVAS. Therefore, upright polypropylene infusion bags are superior to non-PVC infusion bags in leakage rates and economic losses. We hope this study can help to improve the product quality of different infusion packaging materials, reduce drug and clinical economic losses, and reduce the safety hazards of medication.

## Discussion

There is a high proportion of intravenous infusion therapy and a large consumption of regular infusion in China. Therefore, differences in the forms of different regular infusions may result in exponentially amplified differences in clinical experience, medication risks, and economic losses. This study systematically compared the difference in the leakage rate of two types of closed intravenous infusion containers used in PIVAS as well as the causes of leakage and the economic losses due to leakage. We hope to provide references for the selection of intravenous infusion containers in Chinese medical institutions. In this study, PIVAS of 21 regional tertiary hospitals in China were included to ensure that the operations were standardized and the staff had received professional training to minimize the occurrence of leakage caused by the irregular operation of personnel. Before the study, the project leaders of 21 research institutions were trained centrally to ensure that the research was free from human interference and to ensure the consistency of the data. During the 3-month real-world research, a total of 5.6 million data points were collected. This enormous amount of data can reduce the interference of accidental events and ensure the authenticity and reliability of the data.

In total, 2,349,899 upright polypropylene infusion bags and 3,301,722 non-PVC infusion bags were collected. Eleven cases of upright polypropylene infusion bag leakage occurred (with a leakage rate of 0.05‱), and 394 cases of non-PVC infusion bag leakage occurred (with a leakage rate of 1.19‱). The leakage rate of upright polypropylene infusion bags was significantly lower than that of non-PVC infusion bags. With regard to detecting leakage, regardless of the phase of PIVAS, the number and rate of leakages of non-PVC infusion bags were higher than upright polypropylene infusion bags, with significant differences in the comparison. Especially in the pre-PIVAS stage, leakage occurred only in the non-PVC bags. The pre-PIVAS stage includes only the storage and transportation of infusion fluids from the factory to medical institutions. Therefore, non-PVC infusion bags have poorer resistance to pressure and falling resistance during transportation, which may be related to their materials and the heat sealing and welding processes. This result is similar to the results of Chen’s study (Chen et al., 2014), which showed that non-PVC infusion bags are prone to leakage during storage and transport.

With regard to the cause of the leakage, the main reason for leakage from upright polypropylene infusion bags was “sharp objects such as glass fragments or aluminum caps piercing the bag,” which is caused by manual operation. This suggests that the leakage of upright polypropylene infusion bags is mostly related to external reasons rather than their own quality characteristics. However, the main reason for leakage of non-PVC infusion bags was “squeezing, stacking, and uneven arrangement cause folding of edges,” which is unavoidable during infusion use. There was only one case of leakage of an upright polypropylene infusion bag for this reason. Additionally, non-PVC infusion bags have leakage caused by “leakage at the nozzle joint,” “leakage at the joint,” and “valve port quality issues.” This leakage does not occur in upright polypropylene infusion bags, which might be related to their quality characteristics. For the proportion of leakage due to external factors such as “sharp objects such as glass fragments or aluminum caps piercing the bags,” “excessive manual or machine throwing force,” and “falling” for all bags, the leakage of upright polypropylene infusion bags was lower than that of non-PVC infusion bags. This may be because the upright polypropylene infusion bags can be placed upright to reduce the possibility of contact with sharp objects on the operating surface, have higher strength, and have stronger compression and drop resistance. Consequently, hospitals that use non-PVC infusion bags are at greater risk than hospitals that use upright polypropylene infusion bags, especially for Chinese tertiary hospitals with annual infusion volumes in the millions.

The two infusion container materials and processes are different, resulting in different risks of leakage. Non-PVC infusion bags are made of non-PVC composite film by heat welding, and the infusion cover and the bag body are connected by heat welding. Because the heat welding process requires strict control of parameters, minor deviations can lead to poor welding or excessive welding and ultimately cause leakage (Liu et al., 2015). A study found that non-PVC material may be stiff and fragile and may be prone to leakage when used for peritoneal dialysis, resulting in peritonitis (Zhou et al., 2015). In contrast, upright polypropylene infusion bags are made of polypropylene pellets by a hot-melt process, the bag body is formed in one piece without welding points, and the bag body and infusion cover are connected by hot-melt welding, which has high welding strength and therefore strong resistance to falls and pressure (Qi et al., 2017). This might be an important reason why the leakage rate of upright polypropylene infusion bags is lower. In addition, various aspects of the nonstandard operation of non-PVC infusion bags have an impact on leakage, such as extrusion and vibration during storage and transfer; sharps scratches during unpacking; pinpricks, glass fragment scratches, and excessive extrusion during the preparation process; and transport extrusion and pinpricks during infusion use (Li H. et al., 2019). Furthermore, climate, temperature, and physical abrasion all challenge the packaging of non-PVC materials (Zhou et al., 2015).

The economic loss caused by leakage is closely related to whether it is admixed and the types of admixed drugs. Because the leakage rate of non-PVC infusion bags is higher than that of upright polypropylene infusion bags, hospitals that choose to use non-PVC bags have a greater possibility of economic loss caused by leakage. In our study, one case of leakage was found when a non-PVC bag was admixed with thiotepa injection (7 × 10 mg), resulting in an economic loss of 19,063.29 CNY. For hospitals with a regular annual infusion usage of over a million, regular infusion containers with a lower risk of leakage should be chosen to avoid significant economic losses caused by occasional leakage incidents.

Totally, leakage occurred in 136 cases of common drug, 24 cases of antibiotic drug, and 6 cases antineoplastic drug. The drug with the highest frequency of leakage is potassium chloride injection, with a total of 32 occurrence in common drugs. But this drug was relatively safe when it leaked. The types of drugs involved nervous system drugs, cardiovascular system drugs, respiratory system drugs, and so on. Compared to common drug, antineoplastic drug and antibiotic drug pose greater harm to personnel. In antineoplastic drug, the most frequent occurrence of leakage is in carboplatin injection, which can cause huge harm to the human body. It not only has blood toxicity, but also has gastrointestinal toxicity and kidney toxicity. In addition, other antineoplastic drugs that caused leakage, such as aclarubicin, oxaliplatin and thiotepa injection, can also cause toxic to the human body. As for antibiotic drug, the most frequent occurrence of leakage is in meropenem injection, which can lead to allergic reactions and increase the body’s resistance to the drug. Other antibiotic drugs that leak were mainly cephalosporins, which can cause antibiotic contamination in the environment. Therefore, as we can know from the leaking drugs, although the leakage is not caused by the drug, the damage caused by the drug is inevitable, and even some damage is irreversible.

Occupational exposure to drugs and its association with adverse health effects has been well demonstrated over the decades, especially antineoplastic drugs (Yu et al., 2022). Previous studies have demonstrated that the environmental pollution caused by leakage causes physiological damage to PIVAS staff, and long-term occupational exposure also causes injuries to staff, especially for cytotoxic drugs (Connor et al., 2014; Hao et al., 2022). Raveena confirmed that environmental contamination plays a role in biological exposure to cyclophosphamide (Raveena et al., 2015). Some studies focused on antineoplastic drug residue monitoring, for example, ultra-high pressure liquid chromatography separation coupled to tandem mass spectrometry detection (UHPLC-MS/MS) methods for the detection of surface samples (Fleury et al., 2022), ultra-high performance liquid chromatography quadrupole orbitrap high resolution mass spectrometry (UPLC-Q/Orbitrap-HRMS) method for the rapid detection and monitor of 15 cytotoxic drugs in PIVAS (Yu et al., 2022). Other studies focused on the health risks associated with occupational exposures to antineoplastic drugs, and the findings were generally indicative of an increased risk of adverse reproductive outcomes with occupational exposure (Connor et al., 2014). Also, a meta-analysis found a significant association between occupational exposure to antineoplastics drugs and increases in chromosomal aberrations in healthcare workers (Roussel et al., 2019). However, despite following the published guidelines, occupational exposure still exists, exposing healthcare staffs in PIVAS or out of PIVAS to dangers. Therefore, reducing the occurrence of leakage is also an important way to protect the life and health of PIVAS staffs.

Leakage also affects the operation efficiency of staff in PIVAS and increases their workload. When leakage occurs after admixture, it is necessary not only to clean up the leaky drug but also to admix it again. The National Health Commission of the PRC, PIVAS Construction and Management Guidelines note that there are strict procedures for handling drug leakage, especially for harmful drugs (Zhang et al., 2022). Infusion leakage seriously affects the efficiency of personnel operation.

A satisfaction questionnaire was conducted among 259 staff members, approximately 70% of whom had more than 5 years of PIVAS work experience. The findings indicated that the survey results were credible. The long-term use of the two infusion containers was also evaluated by PIVAS staff members. The satisfaction survey results showed that the respondents believed that the upright polypropylene bags were better than the non-PVC bags in terms of fall resistance and pressure resistance scores as well as leakage economy and time loss scores with significant differences. In the long-term work of PIVAS staff, the higher pressure resistance and lower risk of leakage of upright polypropylene infusion bags have been widely recognized. In this study, from the statistical data and the clinical experience of PIVAS staff, we found that the leakage of non-PVC bags was higher than that of upright polypropylene bags.

This study has some limitations. In China, there are still a large number of medical institutions that have not established PIVAS. Intravenous infusions are admixed in outpatient clinics, wards and other places. These scenarios have a higher risk of leakage, and the harm caused by leakage is greater. Follow-up studies should include these locations so that research on the leakage rate of intravenous infusion materials is more complete. It is also recommended that these medical institutions prioritize infusion containers with less risk of leakage.

## Data Availability

The original contributions presented in the study are included in the article/Supplementary Material, further inquiries can be directed to the corresponding authors.
